# Disruption in Brain Phospholipid Content in a Humanized Tau Transgenic Model Following Repetitive Mild Traumatic Brain Injury

**DOI:** 10.3389/fnins.2018.00893

**Published:** 2018-12-04

**Authors:** Joseph O. Ojo, Moustafa Algamal, Paige Leary, Laila Abdullah, Benoit Mouzon, James E. Evans, Michael Mullan, Fiona Crawford

**Affiliations:** ^1^Experimental Neuropathology and Omics Laboratory, Roskamp Institute, Sarasota, FL, United States; ^2^James A. Haley Veterans’ Hospital, Tampa, FL, United States; ^3^The School of Life, Health and Chemical Sciences, Open University, Milton Keynes, United Kingdom

**Keywords:** repetitive mild traumatic brain injury, hTau, cortex, hippocampus, phospholipids, omega-3 and 6 fatty acids, arachidonic acid, docosahexaenoic acid

## Abstract

Repetitive mild traumatic brain injury (mTBI) is a risk factor for the development of neurodegenerative diseases such as chronic traumatic encephalopathy typified by immunoreactive tau aggregates in the depths of the sulci. However, the underlying neurobiological mechanisms involved have not been largely explored. Phospholipids are important molecules which form membrane lipid bilayers; they are ubiquitous to every cell in the brain, and carry out a host of different functions. Imbalance in phospholipid metabolism, signaling and transport has been documented in some neurological conditions. However, not much is currently known about their roles in repetitive mTBI and how this may confer risk for the development of age-related neurodegenerative diseases. To address this question, we designed a longitudinal study (24 h, 3, 6, 9, and 12 months post-injury) to comprehensively investigate mTBI dependent brain phospholipid profiles compared to sham counterparts. We use our established mouse model of repetitive mTBI that has been extensively characterized up to 1-year post-injury in humanized tau (hTau) mice, which expresses all six human tau isoforms, on a null murine background. Our data indicates a significant increase in sphingomyelin, phosphatidylethanolamine (PE), phosphatidylcholine (PC), and derivative lysoPE and lysoPC at acute and/or sub-acute time points post-injury within the cortex and hippocampus. There was also a parallel increase at early time points in monounsaturated, polyunsaturated and saturated fatty acids. Omega-6 (arachidonic acid) to omega-3 (docosahexaenoic acid) fatty acid ratio for PE and PC species was increased also at 24 h and 3 months post-injury in both hippocampus and cortex. The long-term consequences of these early changes in phospholipids on neuronal and non-neuronal cell function is unclear, and warrants further study. Understanding phospholipid metabolism, signaling and transport following TBI could be valuable; they may offer novel targets for therapeutic intervention not only in TBI but other neurodegenerative diseases.

## Introduction

Exposure to a history of repetitive mild traumatic brain injury (mTBI) has been recognized as a major risk factor for the development of age-related degenerative diseases, such as Alzheimer’s disease, and chronic traumatic encephalopathy (CTE) typified by immunoreactive tau aggregates in the depths of the sulci (Gedye et al., 1989; Mortimer et al., 1991; Schofield et al., 1997; Fleminger et al., 2003; McKee et al., 2013; Omalu et al., 2011; Smith et al., 2013). The underlying neurobiological mechanisms that precipitates these disease phenotypes have not been largely explored.

Lipidomic profiling is an extremely powerful tool that enables large-scale study of novel pathways and networks of lipids in biological systems (Sparvero et al., 2010; Wenk, 2010). It has been utilized very successfully in recognizing the roles of lipids in several metabolic diseases such as atherosclerosis, hypertension and diabetes, but to date has received little attention in the study of neurodegenerative disorders. The brain is one of the richest tissues in terms of phospholipid content. Phospholipids are important molecules, forming the membrane lipid bilayers of neurons, glia and cerebrovascular cells. They provide structural integrity for intracellular and cell surface membrane proteins (Adibhatla and Hatcher, 2007). They have a host of diverse roles ranging from regulating behavior of membrane proteins, receptors, enzymes, ion channels, serving as bioenergetics reservoirs and precursors of secondary messengers for signal transduction, including mediating inflammatory responses (Adibhatla and Hatcher, 2007). An imbalance in the coordination of phospholipid metabolism has been well documented in several neurological and psychiatric conditions, resulting in diverse phenotypes and disease states (Kosicek and Hecimovic, 2013). In human severe TBI cases, increases in phospholipid levels from lipoprotein fractions, and free fatty acids in the CSF have been reported within hours to days after injury (Pilitsis et al., 2003; Pasvogel et al., 2010; Hankin et al., 2011). Also severe TBI mouse models involving controlled cortical injury (CCI) show increased levels of phospholipids and their metabolites in the brain at acute to sub-acute time points post-injury (Homayoun et al., 1997, 2000; Abdullah et al., 2014). There are, however, no current studies that have explored the role of phospholipids in repetitive mTBI, especially at chronic time points post-injury, and how this may confer risk for the development of age-related neurodegenerative diseases.

To address this question herein, we designed a prospective and longitudinal study to comprehensively investigate repetitive mTBI dependent and age-related changes in the brain (hippocampus and cortex) phospholipid profiles in a preclinical mouse model. We focus on time points 24 h, 3, 6, 9, and 12 months post-injury to cover the span of acute, sub-acute and chronic changes following TBI. We have chosen to use our previously established mouse model of repetitive mTBI with 5 hits administered to young mice over a 2-week period. This model has been extensively characterized from 24 h to 24 months post-injury. Animals demonstrate persistent deficits in spatial memory, and white matter damage typified by corpus callosum thinning, axonal injury and gliosis (Mouzon et al., 2012; Ojo et al., 2013; Mouzon et al., 2014; Ojo et al., 2015, 2018). Given the involvement of tau in CTE, and our previous reports of TBI dependent tau pathology following TBI in tau transgenic mice (Ojo et al., 2013, 2016; Mouzon et al., 2018a,b,c), we have chosen to use the hTau mice which expresses all six isoforms of human tau, on a null murine background to closely mimic the human condition (Andorfer et al., 2003).

We report in this first study, a significant increase in sphingomyelin, phosphatidylethanolamine (PE), phosphatidylcholine (PC), and derivative lysoPE and lysoPC at acute and/or sub-acute time points post-injury within the cortex and hippocampus. There was also a parallel increase at early time points in monounsaturated, polyunsaturated and saturated fatty acids. The omega-6 (arachidonic acid) to omega-3 (docosahexaenoic acid) fatty acid ratios for PE and PC species were increased also at 24 h and 3 months post-injury in the both hippocampus and cortex. The long-term consequences of these early phospholipid changes on neuronal and non-neuronal cell function is unclear, but warrants further study. Understanding phospholipid metabolism, signaling and transport could be valuable to the study of TBI, as they may offer a novel targets and indicate the appropriate or optimum time frame for therapeutic intervention.

## Materials and Methods

### Animals

Transgenic mice expressing human tau on a C57BL/6 and null murine tau background (generated as previously described by Andorfer et al., 2003) were purchased from Jackson Laboratories, Bar Harbor, ME, United States. Animals were 12 weeks old prior to injury exposure. Animals were housed in standard cages under a 12-h light/12-h dark schedule at ambient temperature controlled between 22 and 23°C under specific pathogen free conditions. Animals were given food and water *ad libitum* and maintained under veterinary supervision throughout the study. There was no evidence of disease among the colony. Male mice were randomly assigned to experimental groups consisting of a sample size of 4 per group. All mice were male to avoid any confounding effects of gender and to limit the numbers of mice required. Experiments were performed in accordance with Office of Laboratory Animal Welfare and National Institutes of Health guidelines under a protocol approved by the Roskamp Institute Institutional Animal Care and Use Committee (IACUC - R054). All analyses were carried out blind to study group assignment.

### Experimental mTBI

The experimental TBI methods were performed as previously described (Mouzon et al., 2012). Briefly, mice were anesthetized with 1.5 L per minute of oxygen and 3% isoflurane for 3 min. After shaving of the injury site, mice were transferred into a stereotaxic frame (Just For Mice Stereotaxic, Stoelting, Wood Dale, IL, United States) mounted with an electromagnetic controlled impact device (Impact One Stereotaxic Motorized Impactor, Richmond, IL, United States). Heads were positioned and fixed in the device, which prevented lateral movements as the impact was delivered. All mice were placed on a heating pad to maintain their body temperature at 37(C. A 5-mm blunt metal impactor tip attached to the electromagnetic motorized device was zeroed on the scalp and positioned above the midsagittal suture before each impact using the NeuroLab controller. On satisfactory positioning, the tip was retracted and the depth was adjusted to the desired level. The scalp was gently stretched by hand to restrict lateralization of the impact and to prevent the rod from delivering an inadequate trauma load at an irregular angle. Injury parameters were 5 m per second strike velocity, 1.0 mm strike depth, 200 milliseconds dwell time, and a force of 72N. This sublethal impact does not cause direct tissue damage to the injury site, and there is no development of skull fracture or subdural hemorrhage, even after repetitive injuries. Mice in the r-TBI group received 5 hits over a 9-day period with an inter-injury interval of 48 hours. Repetitive sham control mice received anesthesias of the same frequency and duration ((˜3 mins per session) as their r-TBI counterparts. After each impact was delivered, the mice were allowed to recover on a heating pad set at 37(C to prevent hypothermia. On becoming ambulatory, mice were returned to their cages and carefully monitored for any abnormalities.

### Lipidomic Analyses

Anesthetized animals at euthanasia were sacrificed by cardiac puncture, and perfused transcardially with phosphate buffer saline solution. Brain tissue was removed and cortices were dissected and flash frozen in liquid nitrogen. Cortices were homogenized in LC/MS grade water in a volume of 2.5× wet weight. Fifty microliter aliquots were stored specifically for lipidomic analysis. The Folch method (Folch et al., 1957) was used to extract lipids from brain samples spiked with synthetic internal standards [di-14:0 FA containing PC and PE, 14:0 FA containing (LPE) and (LPC), d18:1/17:0 SM, and di-16:0 for PI]. Dried lipid extracts were re-suspended in isopropanol and separation was achieved using hydrophilic interaction chromatography (HILIC) on a 1 mm × 100 mm column packed with 3 μm Pinnacle II silica particles (Restek Corporation, Bellefonte, PA, United States). An isocratic run was performed with 70% solvent A [100% acetonitrile (ACN)] in 30% solvent B (78% methanol, 1% formic acid, 0.6% ammonium hydroxide) for 15 min at a flow rate of 55 μl/min with the column temperature at 40°C. Mass spectrometry (MS) was performed with a Thermo LTQ-XL linear ion trap mass spectrometer equipped with a Surveyor HPLC pumping system and Micro AS autosampler (Thermo Fisher, Waltham, MA, United States). Full scan negative ion mass spectra were acquired from m/z 200 to 2,000 with in-source collision induced dissociation (SCID), with relative energies at 15%. All spectra were obtained with a 200 ms maximum ion time and by summing of 5 microscans. Mass spectra were summed over the chromatographic peak for each PL class and spectra (each as a list of m/z versus intensity signal) and were exported from the XCalibur (Thermo Fisher) to Microsoft Excel (Microsoft, Redmond, WA, United States). Files were then uploaded to LipidomeDB online to identify and quantify each PL molecular species using the internal as a reference for each class. The mass of target lipids and abundances of their isotopic variants were calculated from the chemical formula by adding the masses of the formate adduct ions [M+CHO2]- as described in detail by Emmerich et al. (2017). An independent reference sample was added to each run to control for run-to-run variability. All molecular species identified within each PL class were summed to generate total PC, LPC, PE, LPE, SM, and PI concentration values. Each phospholipid class of PC, LPC, PE, LPE, and PI was then separately grouped according to their degree of unsaturation of each molecular species saturated fatty acids (SFA), monounsaturated fatty acids (MUFA), and polyunsaturated fatty acids (PUFA). We also grouped arachidonic acid (AA)-containing lipid species to docosahexaenoic acid (DHA)– and ether containing species of PC, LPC, PE, LPE, and PI as described elsewhere (Emmerich et al., 2017).

### Statistical Plan

We determined differences in TBI or AD groups using ANOVA or χ2 test. Samples were log transformed when parametric assumptions were not met following tests for normality. In cases when transformation was unsatisfactory, non-parametric testing was used for analyses. We performed Principal Component Analysis (PCA) to minimize multicollinearity and to thus achieve dimension reduction, as we have previously described for the analyses of our lipidomic datasets (Abdullah et al., 2012, 2013, 2014). Individual lipids were analyzed by mixed linear modeling (MLM) regression analysis to identify lipids specifically altered by the study treatment. Prior to performing MLM regression analysis on each relevant component of interest (i.e., our outcome measure), we used the Anderson-Rubin method to export uncorrelated scores, whilst adjusting for random (i.e., human) factors, and to assess independent (i.e., diagnostic and replicative) fixed factors. Following analyses using MLM, Fischer’s least significant difference (LSD) correction and the Benjamini–Hochberg (B–H) procedure were used for multiple-test correction, and control of the false discovery rate (set at 0.01) for all comparisons. Analyses was conducted using SPSS version 17 (IBM corporation) and type I error was controlled by setting α at 0.05.

## Results

### Phospholipid Profiles in the Cortex and Hippocampus of a r-mTBI (hTau) Mouse Model

#### Total Phospholipid Levels in the Cortex and Hippocampus of r-mTBI Mice at Longitudinal Timepoints

**FIGURE 1:**
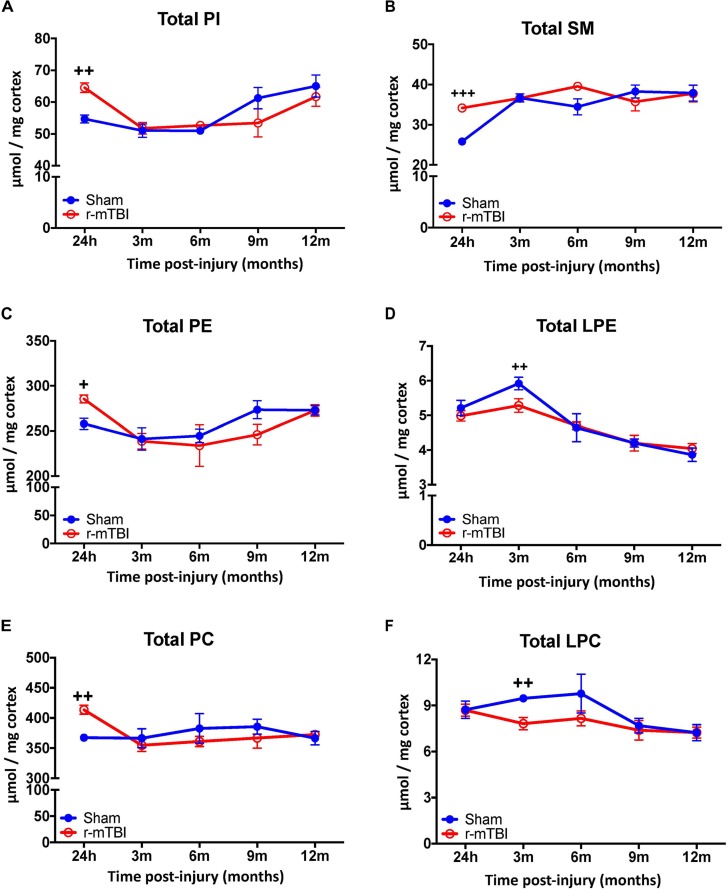
Total Phospholipid levels in the cortex of r-mTBI hTau mice. Significant changes in total phospholipid [PI **(A)**, SM **(B)**, PE **(C)**, LPE **(D)**, PC **(E)**, LPC **(F)**] species in the cortex of a model of repetitive-mTBI in hTau mice. Sample size for all groups across all time points is *n* = 4. All data represent mean μM per (10 mg) wet weight ±SEM. Individual molecular lipid species were quantified by liquid chromatography/mass spectrometry and were summed after LipidomeDB analyses to generate total phospholipid levels. Asterisks represent ^+^*P* < 0.05, ^++^*P* < 0.01, ^+++^*P* < 0.001 for comparisons between sham/r-mTBI mice. PE, phosphatidylethanolamine; LPE, lysophosphatidylethanolamine; PC, phosphatidylcholine; LPC, lysophosphatidylcholine; PI, phosphatidylinositol; SM, sphingomyelin.

The cortex and hippocampus are region that demonstrates significant pathobiological changes in both TBI and AD pathogenesis. We therefore analyzed different total phospholipid species in this brain region of our mouse model. In the cortex, total phosphatidylinositol (PI), PE, PC and sphingomyelin (SM) were significantly increased at 24 h post-injury in r-mTBI mice compared to sham counterparts (Figures 1A–C,E). Total lysophosphatidylethanolamine (LPE) and lysophosphatidylcholine (LPC) were decreased at 3 months post-injury compared to shams their counterparts (Figures 1D,F).

**FIGURE 2:**
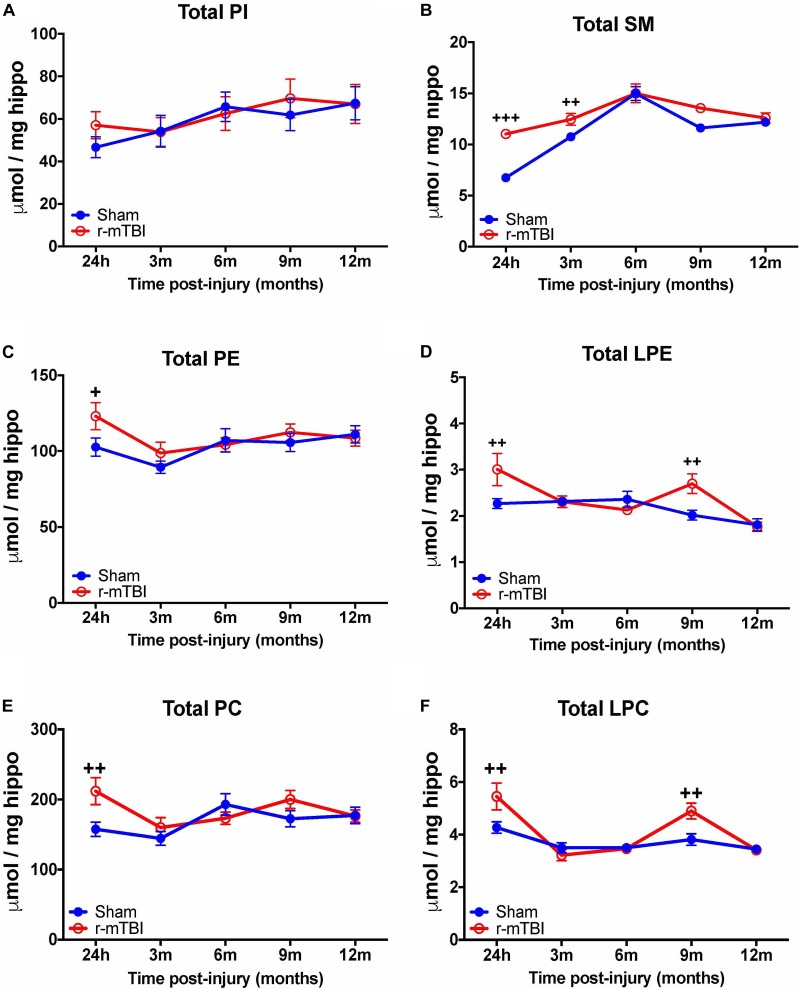
Total Phospholipid levels in the hippocampus of r-mTBI hTau mice. Significant changes in total phospholipid [PI **(A)**, SM **(B)**, PE **(C)**, LPE **(D)**, PC **(E)**, LPC **(F)**] species in the hippocampus of a model of repetitive-mTBI in hTau mice. Sample size for all groups across all time points is *n* = 4. All data represent mean μM per (5.5 mg) wet weight ±SEM. Individual molecular lipid species were quantified by liquid chromatography/mass spectrometry and were summed after LipidomeDB analyses to generate total phospholipid levels. Asterisks represent ^+^*P* < 0.05, ^++^*P* < 0.01, ^+++^*P* < 0.001 for comparisons between sham/r-mTBI mice. PE, phosphatidylethanolamine; LPE, lysophosphatidylethanolamine; PC, phosphatidylcholine; LPC, lysophosphatidylcholine; PI, phosphatidylinositol; SM, sphingomyelin.

Examination of total phospholipid levels in the hippocampus, also revealed a significant increase in SM (at 24 h and 3 months post-injury – Figure 2B), PE and PC (at 24 h post-injury – Figures 2C,E), in repetitive mTBI mice compared to sham counterparts. However, unlike changes observed in the cortex, we observed a significant increase in LPE and LPC (at 24 h and 9 months post-injury – Figures 2D,F) in repetitive mTBI mice compared to sham counterparts. Additionally, no change was observed in total PI in the hippocampus of r-mTBI mice vs. Sham counterparts at all timepoints examined (Figure 2A).

#### Total Ether PE and Ether PC Levels in the Cortex and Hippocampus of Repetitive-mTBI Mice at Longitudinal Timepoints

**FIGURE 3:**
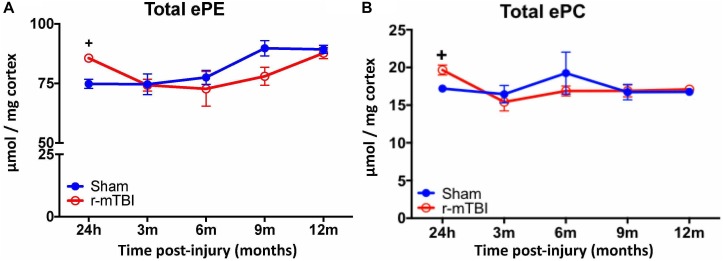
Total ether PE and ether PC levels in the cortex of repetitive-mTBI hTau mice. Significant changes in total etherPE **(A)** and etherPC **(B)** lipid species in the cortex of a model of repetitive-mTBI in hTau mice. Sample size for all groups across all time points is *n* = 4. All data represent mean μM per (10 mg) wet weight ±SEM. Individual molecular lipid species were quantified by liquid chromatography/mass spectrometry and were summed after LipidomeDB analyses to generate total etherphospholipid levels. Asterisks represent ^+^*P* < 0.05 for comparisons between sham/r-mTBI mice. ePE, etherphosphatidylethanolamine; ePC, etherphosphatidylcholine.

**FIGURE 4:**
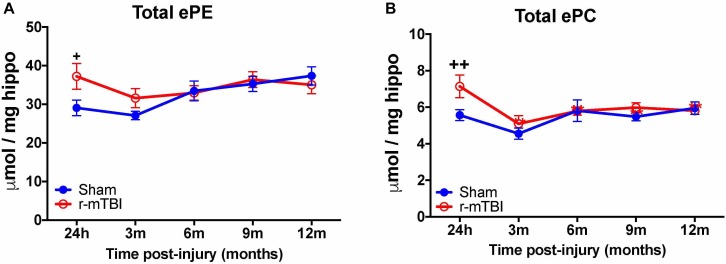
Total etherPE and ether PC levels in the hippocampus of repetitive-mTBI hTaumice. Significant changes in total etherPE **(A)** and etherPC **(B)** lipid species in the hippocampus etc. of a model of repetitive-mTBI in hTau mice. Sample size for all groups across all time points is *n* = 4. All data represent mean μM per (5.5 mg) wet weight ±SEM. Individual molecular lipid species were quantified by liquid chromatography/mass spectrometry and were summed after LipidomeDB analyses to generate total etherphospholipid levels. Asterisks represent ^+^*P* < 0.05, ^++^*P* < 0.01 for comparisons between sham/r-mTBI mice. ePE, etherphosphatidylethanolamine; ePC, etherphosphatidylcholine.

Given the changes observed in both PE and PC species, we therefore proceeded to analyze the derivatives of total PE and PC in the cortices and hippocampi of our TBI models as they are physiologically relevant phospholipid species which impact on brain function. In the cortex, there was a significant increase in total ether-phosphatidylethanolamine (ePE) and ether-phosphatidylcholine (ePC) at 24 h post-injury in r-mTBI mice vs. shams (Figures 3A,B). Similarly, investigation of hippocampal homogenates revealed a significant increase in total ether-phosphatidylethanolamine (ePE) and ether-phosphatidylcholine (ePC) at the 24 h post-injury time point in r-mTBI mice vs. shams (Figures 4A,B).

#### Arachidonic Acid (AA) and Docosahexaenoic Acid (DHA) Containing Phospholipid Species, and Their Ratio in the Cortex and Hippocampus of Repetitive mTBI Mice at Longitudinal Timepoints

**FIGURE 5:**
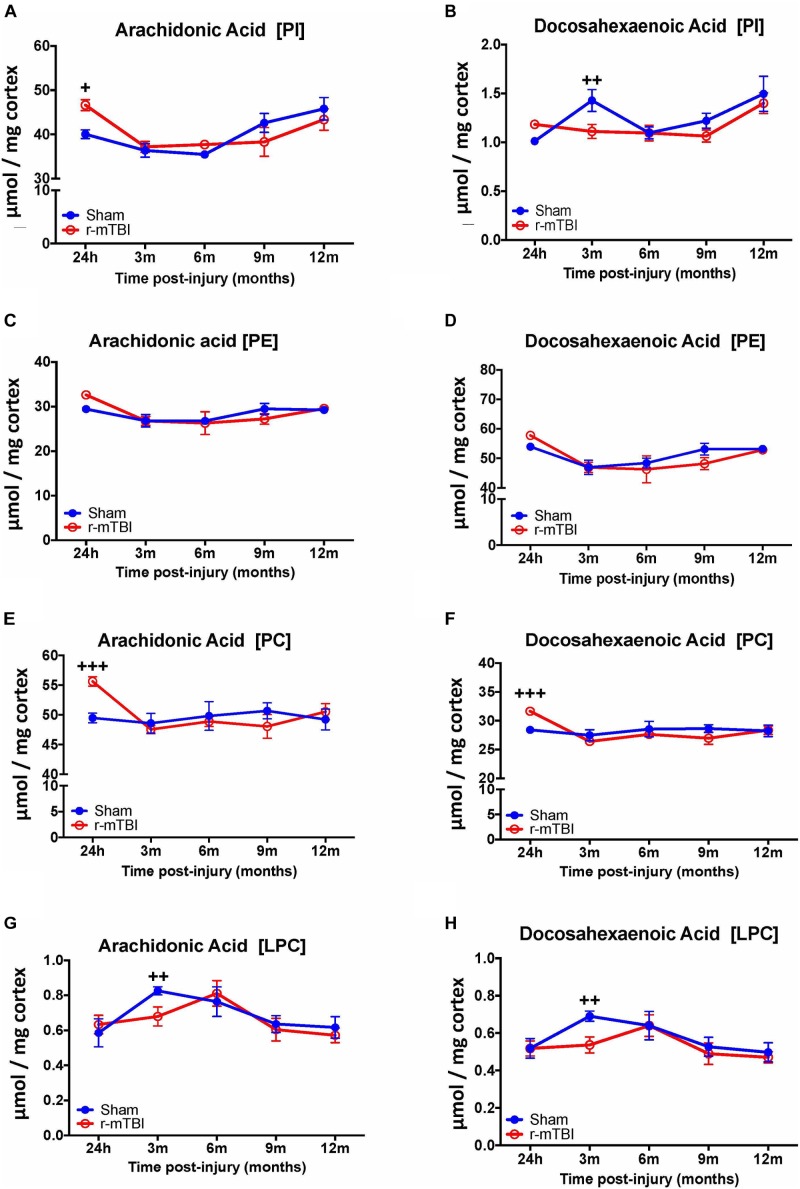
Arachidonic acid and docosahexaenoic acid containing phospholipid species in the cortex of repetitive mTBI hTau mice. Significant changes in arachidonic acid and docosahexaenoic acid containing PI **(A,B)**, PE **(C,D)**, PC **(E,F)**, and LPC **(G,H)** species in the cortex of a model of repetitive-mTBI in hTau mice. Sample size for all groups across all time points is *n* = 4. Data represents mean μmol per wet weight (10 mg) ±SEM. Individual molecular lipid species were quantified by liquid chromatography/mass spectrometry and were summed after LipidomeDB analyses to generate arachidonic and docosahexaenoic acid levels for each phospholipid species. Asterisks represent ^+^*P* < 0.05, ^++^*P* < 0.01, ^+++^*P* < 0.001 for comparisons between sham/r-mTBI mice. PE, phosphatidylethanolamine; PC, phosphatidylcholine; PI, phosphatidylinositol.

In the cortex, we observed a significant increase in AA levels for PI and PC species at the 24 h post-injury time point in the repetitive mTBI vs. sham mice (Figures 5A,E), and a significant decrease in AA levels for LPC was also observed at the 3 months post-injury time point in the repetitive mTBI vs. sham mice (Figure 5G). DHA levels for PI and LPC in the cortex were significantly decreased at 3 months post-injury in repetitive-mTBI compared to aged matched sham mice (Figures 5B,H), however, there was an increase in DHA levels for PC at 24 h post-injury in the repetitive mTBI vs. sham mice (Figure 5F). No change was observed for AA and DHA levels for PE species in repetitive mTBI vs. sham mice (Figures 5C,D).

**FIGURE 6:**
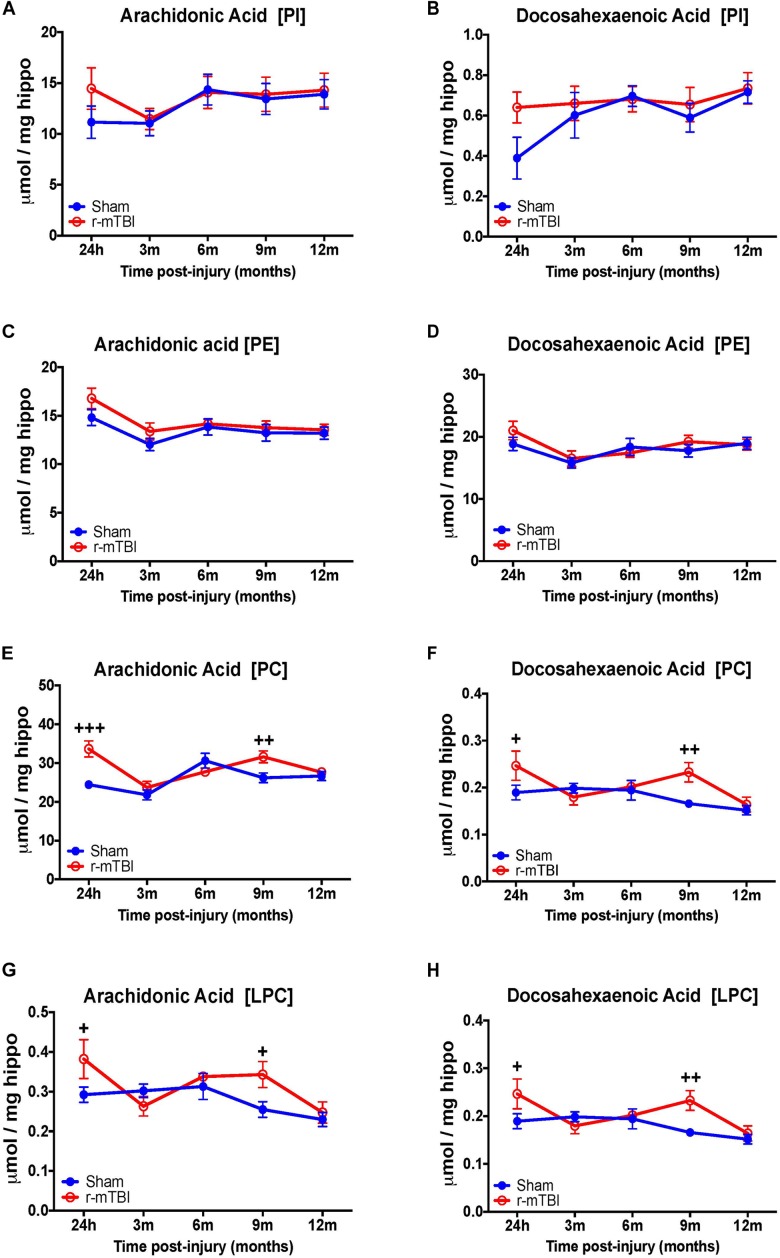
Arachidonic acid and docosahexaenoic acid containing phospholipid species in the hippocampus of repetitive mTBI hTau mice. Significant changes in arachidonic acid and docosahexaenoic acid containing PI **(A,B)**, PE **(C,D)**, PC **(E,F)**, and LPC **(G,H)** species in the hippocampus of a model of repetitive-mTBI in hTau mice. Sample size for all groups across all time points is *n* = 4. Data represent mean μmol per wet weight (5.5 mg) ±SEM. Individual molecular lipid species were quantified by liquid chromatography/mass spectrometry and were summed after LipidomeDB analyses to generate arachidonic and decosahexaenoic acid levels for each phospholipid species. Asterisks represent ^+^*P* < 0.05, ^++^*P* < 0.01, ^+++^*P* < 0.001 for comparisons between sham/r-mTBI mice. PE, phosphatidylethanolamine; PC, phosphatidylcholine; PI, phosphatidylinositol; LPC, lysophosphatidylcholine.

Our interrogation of the hippocampus revealed a similar changes observed in the cortex for AA and DHA levels for PC species at 24 h, including also at 9 months post-injury time points in repetitive mTBI vs. sham mice, but diverging effects were observed in the hippocampus for AA and DHA levels for LPC typified by a significant increase also at 24 h and 9 months post-injury in repetitive mTBI vs. sham mice (Figures 6E–H). We did not observe any change for AA and DHA levels for both PI and PE species in the hippocampus in repetitive mTBI vs. sham mice at any of the time points examined (Figures 6A–D).

Arachidonic acid (AA) and docosahexaenoic acid (DHA) are polyunsaturated fatty acids, with prominent pro-inflammatory and anti-inflammatory potential in the brain milieu, respectively. We thus analyzed the levels of AA to DHA containing phospholipid species (namely PE, PC, LPC, and PI) and their relative ratio in both of our models. Notably, we observed a significant increase in the AA to DHA ratio for PE and PI species in the cortex at 24 h and 3 months, respectively, following repetitive mTBI compared to shams (Table 1), with no change in AA to DHA ratio for PC or LPC observed following any of the post-injury timepoints we examined (Table 1).

**Table 1:** Polyunsaturated fatty acid containing phospholipid species and arachidonic acid to docosahexaenoic acid ratio in the cortex of repetitive mTBI hTau mice.

		Polyunsaturated fatty acid containing lipid species															
		
		Phosphatidylcholine (PC)				Phosphatidylethanolamine (PE)				Phosphatidylinositol (PI)				Lysophosphatidylcholine (LPC)			
					
		PUFA		AA to DHA ratio		PUFA		AA to DHA ratio		PUFA		AA to DHA ratio		PUFA		AA to DHA ratio	
									
		Mean	SEM	Mean	SEM	Mean	SEM	Mean	SEM	Mean	SEM	Mean	SEM	Mean	SEM	Mean	SEM
	24H	107.658	1.529	1.743	0.014	196.096	4.875	0.546	0.003	52.232	1.229	39.698	0.672	1.668	0.249	1.122	0.101
	3M	105.477	3.827	1.770	0.016	183.554	9.720	0.571	0.003	48.719	2.025	27.743	2.879	2.206	0.152	1.204	0.029
SHAM	6M	113.526	9.569	1.746	0.015	187.340	6.213	0.554	0.004	48.254	0.437	33.317	1.720	1.936	0.263	1.208	0.022
	9M	110.043	2.299	1.770	0.017	210.230	7.798	0.554	0.004	58.315	3.229	35.581	1.955	1.819	0.234	1.229	0.029
	12M	108.021	3.667	1.743	0.012	209.777	3.985	0.550	0.003	61.830	3.377	33.970	2.783	1.706	0.203	1.244	0.010
	24H	120.880^+++^	1.800	1.758	0.009	216.893	3.416	0.565^++^	0.004	61.180^++^	1.476	39.486	0.672	1.664	0.179	1.222	0.025
	3M	102.138	1.074	1.801	0.016	182.042	6.539	0.571	0.004	49.287	1.709	34.407^+^	1.342	1.764	0.182	1.268	0.021
r-mTBI	6M	106.410	2.022	1.769	0.011	178.112	17.649	0.569	0.004	50.289	0.939	35.988	2.177	2.079	0.251	1.270	0.021
	9M	104.953	4.297	1.781	0.010	188.896	8.696	0.565	0.003	50.972	4.222	35.590	1.500	1.645	0.244	1.246	0.029
	12M	109.668	2.492	1.781	0.006	208.806	4.911	0.560	0.003	58.471	2.978	32.618	2.728	1.587	0.173	1.207	0.019


*Significant changes in polyunsaturated fatty acid containing PC, PE, PI, and LPC species, and arachidonic acid to docosahexaenoic acid ratio for the same species in the cortex of repetitive mTBI hTau mice. Sample size for all groups across all time points is n = 4. All data represent mean μM per (10 mg) wet weight ±SEM. Individual molecular lipid species were quantified by liquid chromatography/mass spectrometry and were summed after LipidomeDB analyses to generate polyunsaturated fatty acid classification for each phospholipid species, and a ratio of arachidonic acid to docosahexaenoic acid was compiled from these values. Asterisks represent ^+^P < 0.05, ^++^P < 0.01, ^+++^P < 0.001 for comparisons between sham/r-mTBI mice. PE, phosphatidylethanolamine; PC, phosphatidylcholine; PI, phosphatidylinositol; AA, arachidonic acid; DHA, docosahexaenoic acid.*

In the hippocampus, we observed a significant increase in AA to DHA ratio for PC species at 24 h, 3 and 6 months post-injury, and also for PE species an increase at 3 months post-injury time point in repetitive mTBI compared to sham mice (Table 2). No changes in the AA to DHA ratio for PI and LPC species were observed at any of the time points examined (Table 2).

**Table 2:** Polyunsaturated fatty acid containing phospholipid species and arachidonic acid to decosahexaenoic acid ratio in the cortex of repetitive mTBI hTau mice.

		Polyunsaturated fatty acid containing lipid species															
		
		Phosphatidylcholine (PC)				Phosphatidylethanolamine (PE)				Phosphatidylinositol (PI)				Lysophosphatidylcholine (LPC)			
					
		PUFA		AA to DHA ratio		PUFA		AA to DHA ratio		PUFA		AAto DHA ratio		PUFA		AA to DHA ratio	
									
		Mean	SEM	Mean	SEM	Mean	SEM	Mean	SEM	Mean	SEM	Mean	SEM	Mean	SEM	Mean	SEM
SHAM	24H	44.616	2.004	2.453	0.020	75.847	4.508	0.785	0.009	36.822	3.381	25.241	5.777	0.482	0.035	1.578	0.083
	3M	39.129	2.120	2.524	0.032	65.419	3.023	0.762	0.007	41.829	5.561	25.125	5.101	0.501	0.028	1.525	0.045
	6M	54.141	3.413	2.566	0.026	77.763	5.682	0.764	0.016	52.246	4.816	23.129	3.963	0.507	0.054	1.617	0.030
	9M	47.326	2.023	2.547	0.029	77.745	4.420	0.744	0.013	49.809	5.576	24.035	3.805	0.421	0.027	1.522	0.061
	12M	49.099	2.192	2.449	0.021	81.438	4.348	0.699	0.010	55.002	5.912	21.718	3.391	0.381	0.028	1.521	0.068
r-mTBI	24H	59.814^+^	3.993	2.593^+^	0.030	89.207^+^	6.675	0.804	0.009	44.042	4.283	25.978	5.025	0.629^+^	0.080	1.578	0.055
	3M	42.445	2.741	2.658^+^	0.056	72.949	5.303	0.819^+^	0.019	42.437	4.946	26.560	7.145	0.443	0.041	1.464	0.043
	6M	49.112	1.564	2.677^+^	0.020	76.329	3.393	0.817	0.006	49.403	5.791	24.294	4.354	0.540	0.017	1.685	0.040
	9M	56.457	2.584	2.540	0.014	81.952	4.211	0.719	0.006	56.038	6.935	26.579	4.517	0.576	0.053	1.470	0.035
	12M	50.088	1.564	2.480	0.022	79.590	4.097	0.725	0.009	54.007	7.053	22.781	4.104	0.411	0.043	1.509	0.061


*Significant changes in polyunsaturated fatty acid containing PC, PE, PI, and LPC species, and arachidonic acid to decosahexaenoic acid ratio for the same species in the hippocampus of repetitive mTBI hTau mice. Sample size for all groups across all time points is n = 4. All data represent mean μM per (5.5 mg) wet weight ±SEM. Individual molecular lipid species were quantified by liquid chromatography/mass spectrometry and were summed after LipidomeDB analyses to generate polyunsaturated fatty acid classification for each phospholipid species, and a ratio of arachidonic acid to decosahexaenoic acid was compiled from these values. Asterisks represent ^+^P < 0.01 for comparisons between sham/r-mTBI mice. PE, phosphatidylethanolamine; PC, phosphatidylcholine; LPC, lysophosphatidylcholine; PI, phosphatidylinositol; AA, arachidonic acid; DHA, decosahexaenoic acid.*

#### Polyunsaturated, Monounsaturated and Saturated Fatty Acid Containing Phospholipid Species in the Cortex and Hippocampus of Repetitive mTBI Mice

The degree of fatty acid (FA) saturation can influence the function of FA in a variety of ways, thus we proceeded to interrogate monounsaturated, polyunsaturated and saturated fatty acid containing phospholipid species in the cortices and hippocampi of our mouse models. In the cortex, polyunsaturated fatty acid (PUFA) containing PC and PI were significantly increased at 24 h post-injury timepoint in repetitive mTBI compared to sham mice (Table 1), with no changes were observed for PUFA containing PE or LPC following repetitive mTBI (Table 1). In the hippocampus, PUFA containing PC, PE and LPC were significantly increased at 24hrs post-injury in repetitive mTBI compared to sham mice (Table 2).

In the cortex, monounsaturated fatty acid (MUFA) containing PC, PE, PI, and SM were significantly increased at 24 h post-injury in repetitive mTBI compared to sham mice (Table 3), while in the hippocampus, MUFA containing PC and PE were significantly increased at 24 h post-injury, and MUFA containing SM was significantly increased at both 24 h and 3 months post-injury in repetitive mTBI compared to sham mice (Table 4).

**Table 3:** Monounsaturated fatty acid containing phospholipid species in the cortex of repetitive mTBI hTau mice.

		Monounsaturated fatty acid containing lipid species							
		
		Phosphatidylcholine (PC)		Phosphatidylethanolamine (PE)		Phosphatidylinositol (PI)		Sphingomyelin (SM)	
					
		Mean	SEM	Mean	SEM	Mean	SEM	Mean	SEM
	24H	149.247	3.135	22.347	0.450	1.636	0.048	3.586	0.130
	3M	152.515	7.635	22.027	0.999	1.471	0.115	6.959	0.623
SHAM	6M	158.090	10.206	22.682	0.622	1.782	0.123	6.116	0.484
	9M	164.483	6.967	25.139	0.785	1.732	0.176	6.779	0.307
	12M	153.752	4.217	25.161	0.588	2.133	0.113	6.542	0.373
	24H	169.37^+++^	3.328	25.397^+^	0.330	2.251^++^	0.080	4.925^++^	0.222
	3M	149.444	5.736	21.648	0.668	1.516	0.079	6.717	0.214
r-mTBI	6M	149.670	4.397	21.133	2.005	1.538	0.102	7.087	0.089
	9M	154.273	8.622	22.426	1.108	1.634	0.125	6.000	0.447
	12M	154.352	2.581	25.300	0.815	2.089	0.093	6.271	0.383


*Significant changes in monounsaturated fatty acid containing PC, PE, PI, and SM species in the cortex of repetitive mTBI hTau mice. Sample size for all groups across all time points is n = 4. All data represent mean μM per (10 mg) wet weight ±SEM. Individual molecular lipid species were quantified by liquid chromatography/mass spectrometry and were summed after LipidomeDB analyses to generate monounsaturated fatty acid classification for each phospholipid species was compiled from these values. Asterisks represent ^+^P < 0.05, ^+++^P < 0.01, ^+++^P < 0.001 for comparisons between sham/r-mTBI mice. PE, phosphatidylethanolamine; PC, phosphatidylcholine; PI, Phosphatidylinositol; SM, sphingomyelin.*

**Table 4:** Monounsaturated fatty acid containing phospholipid species in the cortex of repetitive mTBI hTau mice.

		Monounsaturated fatty acid containing lipid species							
		
		Phosphatidylcholine (PC)		Phosphatidylethanolamine (PE)		Phosphatidylinositol (PI)		Sphingomyelin (SM)	
					
		Mean	SEM	Mean	SEM	Mean	SEM	Mean	SEM
SHAM	24H	63.673	4.493	8.923	0.544	5.522	0.933	1.024	0.093
	3M	61.204	4.354	8.723	0.289	6.531	1.065	1.673	0.114
	6M	82.151	7.063	10.935	0.767	7.120	1.100	2.272	0.112
	9M	74.872	5.334	10.827	0.472	6.733	1.094	1.910	0.076
	12M	76.644	5.844	11.771	0.578	6.691	1.021	2.009	0.099
r-mTBI	24H	88.344^+^	8.963	12.035^+^	0.761	7.443	1.241	1.509^+^	0.073
	3M	69.087	6.618	9.672	0.694	6.402	1.099	2.051^+^	0.134
	6M	73.181	4.264	10.459	0.516	7.099	1.217	2.364	0.193
	9M	84.734	5.944	11.883	0.468	7.385	1.191	2.165	0.079
	12M	74.950	4.091	11.173	0.534	6.943	1.207	2.049	0.131


*Significant changes in monounsaturated fatty acid containing PC, PE, PI, and SM species in the hippocampus of repetitive mTBI hTau mice. Sample size for all groups across all time points is n = 4. All data represent mean μM per (5.5 mg) wet weight ±SEM. Individual molecular lipid species were quantified by liquid chromatography/mass spectrometry and were summed after LipidomeDB analyses to generate monounsaturated fatty acid classification for each phospholipid species was compiled from these values. Asterisks represent ^+^P < 0.01 for comparisons between sham/r-mTBI mice. PE, phosphatidylethanolamine; PC, phosphatidylcholine; PI, phosphatidylinositol; SM, sphingomyelin.*

Interrogation of the cortical tissue revealed that saturated fatty acid (SFA) containing PC, PI, and SM were significantly increased at 24 h post-injury in repetitive mTBI compared to sham mice (Table 5), while SFA containing PE species were unchanged following injury (Table 5).

**Table 5:** Saturated fatty acid containing phospholipid species in the cortex of repetitive mTBI hTau mice.

		Saturated fatty acid containing lipid species							
		
		Phosphatidylcholine (PC)		Phosphatidylethanolamine (PE)		Phosphatidylinositol (PI)		Sphingomyelin (SM)	
					
		Mean	SEM	Mean	SEM	Mean	SEM	Mean	SEM
SHAM	24H	109.539	2.198	41.258	0.993	0.837	0.024	3.118	0.111
	3M	107.633	4.471	37.153	1.793	0.853	0.051	3.841	0.202
	6M	110.241	5.261	36.196	0.822	0.961	0.030	3.597	0.138
	9M	110.233	4.062	39.737	1.581	1.216	0.231	4.096	0.172
	12M	103.763	3.476	39.541	0.724	1.082	0.038	4.009	0.183
r-mTBI	24H	122.238^++^	2.456	45.013	0.756	1.099^++^	0.036	3.897^++^	0.096
	3M	102.358	3.982	36.492	1.472	0.972	0.173	3.644	0.136
	6M	104.050	2.154	35.985	3.534	0.841	0.079	3.964	0.122
	9M	106.649	4.923	36.164	1.633	0.845	0.052	3.807	0.265
	12M	107.599	1.448	40.187	0.867	1.145	0.052	4.189	0.133


*Significant changes in saturated fatty acid containing PC, PI, PE, and SM species in the cortex of repetitive mTBI hTau mice. Sample size for all groups across all time points is n = 4. All data represent mean μM per (10 mg) wet weight ±SEM. Individual molecular lipid species were quantified by liquid chromatography/mass spectrometry and were summed after LipidomeDB analyses to generate saturated fatty acid classification for each phospholipid species was compiled from these values. Asterisks represent ^++^P < 0.01 for comparisons between sham/r-mTBI mice. PE, phosphatidylethanolamine; PC, phosphatidylcholine; PI, phosphatidylinositol; SM, sphingomyelin.*

In the hippocampus, similar changes were observed for SFA and MUFA containing PC, PE, and SM species. We observed a significant increase in SFA containing PC and PE at 24 h post-injury in repetitive mTBI compared to sham mice (Table 6). SFA containing SM were significantly increased at 24 h and 3 months post-injury in repetitive mTBI compared to sham mice (Table 6). However, no significant change was observed for PUFA, MUFA, or SFA containing PI species following repetitive mTBI (see Tables 2, 4, 6).

**Table 6:** Saturated fatty acid containing phospholipid species in the cortex of repetitive mTBI hTau mice.

		Saturated fatty acid containing lipid species							
		
		Phosphatidylcholine (PC)		Phosphatidylethanolamine (PE)		Phosphatidylinositol (PI)		Sphingomyelin (SM)	
					
		Mean	SEM	Mean	SEM	Mean	SEM	Mean	SEM
SHAM	24H	48.923	3.813	17.882	1.021	4.308	0.761	5.707	0.294
	3M	43.756	3.855	15.200	0.813	5.873	1.091	9.066	0.324
	6M	56.077	5.114	18.305	1.284	6.368	1.077	12.695	0.564
	9M	49.840	4.353	17.084	1.042	5.300	0.856	9.682	0.152
	12M	51.046	3.970	17.834	0.916	5.724	0.927	10.149	0.266
r-mTBI	24H	63.091^+^	6.358	21.793^+^	1.521	5.565	0.971	9.492^+^	0.212
	3M	47.998	5.000	16.071	1.221	5.004	0.875	10.384^+^	0.462
	6M	50.444	3.149	17.430	0.749	5.997	0.992	12.606	0.727
	9M	58.297	4.682	18.543	0.960	6.223	1.025	11.370	0.333
	12M	50.875	3.476	17.780	0.777	6.071	1.036	10.510	0.406


*Significant changes in saturated fatty acid containing PC, PE, PI, and SM species in the hippocampus of repetitive mTBI hTau mice. Sample size for all groups across all time points is n = 4. All data represent mean μM per (5.5 mg) wet weight ±SEM. Individual molecular lipid species were quantified by liquid chromatography/mass spectrometry and were summed after LipidomeDB analyses to generate saturated fatty acid classification for each phospholipid species was compiled from these values. Asterisks represent ^+^P < 0.01 for comparisons between sham/r-mTBI mice. PE, phosphatidylethanolamine; PC, phosphatidylcholine; PI, phosphatidylinositol; SM, sphingomyelin.*

See Supplementary Tables S1–S5 for changes in individual molecular species for each phospholipid class (PC, PE, LPE, PI, and SM) in the cortex of our r-mTBI hTau mouse model.

See Supplementary Tables S6–S10 for changes in individual molecular species for each phospholipid class (PC, LPC, PE, LPE, PI, and SM) in the hippocampus of our r-mTBI hTau mouse model.

## Discussion

We previously demonstrated in a moderate to severe-TBI mouse model (CCI) the involvement of several phospholipid species in the pathogenesis of TBI, 3 months post-injury (Abdullah et al., 2014). At the outset of this project we sought to further investigate the role of phospholipids in repetitive mTBI in a larger and comprehensive study. To address this, we have used a lipidomic approach in a longitudinal study involving five extended time points (ranging from 24 h to 12 months post-injury) following repetitive mild injury in our preclinical mouse model. Such an extended chronic time point evaluation has not been previously explored, and is important in understanding the underlying neurobiological sequelae of TBI pathogenesis. The behavioral and histopathological outcomes in our established preclinical mouse model have been previously characterized at acute (24 h) to chronic (12 months) time points post-injury. Hallmark features include: persistent white matter changes, typified by corpus callosum thinning, glial activation, and deficits in spatial learning and memory (Mouzon et al., 2012, 2014). Given the involvement of tau protein as a key player in the human pathogenesis of repetitive mTBI (Omalu et al., 2011; McKee et al., 2013), we have chosen to use hTau mice, which express all six human tau isoforms on a null murine background to closely mimic the human condition (Andorfer et al., 2003). Emerging neurological and histopathological features have been well characterized in these mice. Human Tau mice progressively develop hyperphosphorylated and aggregated tau as early as 9 months and by 15 months of age show evidence of thioflavine-s positive neurofibrillary tangles. Cognitive deficits in normal object recognition and Morris water maze test appear around 12 months of age and get progressively worse (Andorfer et al., 2003; Polydoro et al., 2009). We have thus chosen to explore timepoints encompassing pre-, peri- and post-“onset” of the cognitive and neuropathological phenotypes, and timepoints post-injury (i.e., 24 h, 3, 6, 9, and 12 months) that capture the anticipated responses overlapping with AD pathogenic changes. Animals were exposed to their injuries at young adulthood (approximately 3 months of age), prior to the emergence of neuropathological features, as this is the vulnerable age-group (e.g., athletes involved in contact sports and military personnel) that we are attempting to mimic who are at risk of exposures to repetitive injuries. hTau mice exposed to our repetitive concussive injury paradigms show similar pathology to our wild-type mice, including evidence of injury dependent acute (early and transient) changes in phospho-tau pathology, without any appreciable chronic and persistent tau pathology 1 year after injury (Mouzon et al., 2012, 2014, 2018a,b). To date the influence of human tau genetic background on brain phospholipids, especially in the context of repetitive mTBI remains elusive.

**FIGURE 7:**
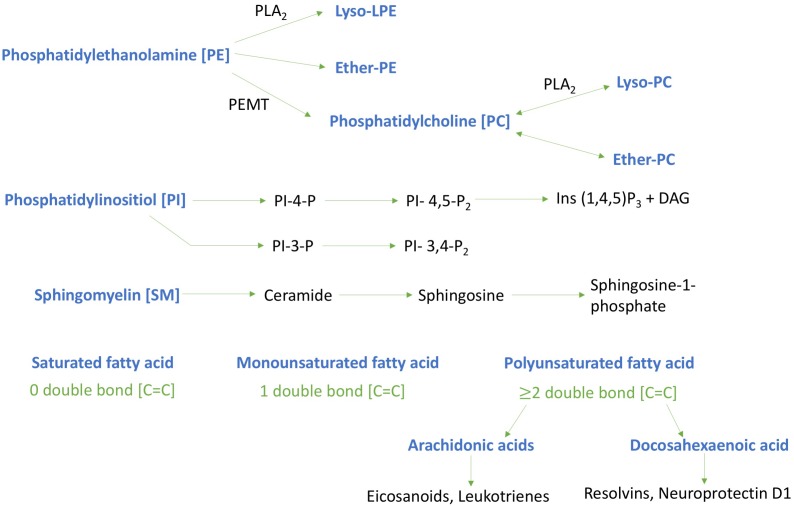
Schematic diagram of key phospholipids investigated and their metabolites. Lipids investigated are highlighted in blue typescript. Phosphatidylinositol - 4 – phosphate [PI-4-P]; Phosphatidylinositol – 4, 5- bisphosphate [PI-4,5-P2]; Inositol trisphosphate [Ins (1,4,5)P3]; Diglyceride [DAG]; Phosphatidylethanolamine *N*-methyltransferase [PEMT]; Phospholipase A2 [PLA2]; Phosphatidylinositol – 3, 4- bisphosphate [PI- 3,4-P2]; Phosphatidylinositol -3- bisphosphate [PI-3-P].

We have focused our investigations herein on six major types of phospholipids: PC, lysophosphatidylcholine (LPC), PI, PE, lysophosphatidylethanolamine (LPE) and sphingomyelin (SM) – see Figure 7.

In the cortex and hippocampus, we report a significant increase in PC and PE at the acute (24 h) time point alone. Tau (human) which is found in membrane rich neuronal structures has been previously shown to interact with the inner leaflet of phospholipid plasma membranes in different *in vitro* models; these phospholipids can modulate tau phosphorylation, facilitate tau aggregation and secretion by *trans*-cellular spreading *in vivo*, and may serve as a pathway for tau aggregates to exert toxicity (Baudier and Cole, 1987; Shea, 1997; Katsinelos et al., 2018). In human AD brains, tangle bearing neurons have been shown to demonstrate increased immunoreactivity for lipid rafts associated proteins derived from lysosomes, PC levels have also been observed in purified paired helical filaments, and given the involvement of tau in transport of vesicles along microtubule tracks, suggests their role in altered subcellular vesicular transport of lipids (Girardot et al., 2003; Gellermann et al., 2006).

We postulate that the increase we observed in phospholipid levels in our model could thus indicate an increased propensity for tau aggregation after repetitive concussions, which appears to occur very early (and transiently) in our model, albeit without other influencing factors *in vivo* (Mouzon et al., 2018a,b). Persistent and chronic tau pathology following TBI in young mice is not a feature of this injury paradigm, however, this has been observed in aged mice using the same injury model (Ojo et al., 2013), and also in a chronic repetitive mTBI paradigm involving 32 hits over 3 months (Ojo et al., 2016), and we thus anticipate that we will observe more prominent changes in the brain lipid profiles of these models. Although no changes in PC and PE were evident between 3 and 12 months post-injury, we did observe a notable decrease in both their derivatives, lysoPE and lyso PC, respectively, at 3 months post-injury in the cortex, and an increase at 24 h and 9 months in the hippocampus of injured mice. This suggests that the early changes in the cortex may primarily be a consequence of injury, whilst the more chronic alterations in the hippocampus may be secondary and related to inflammation. With respect to the latter lysoPC is a strong pro-inflammatory mediator that can act via platelet activating factor (PAF) receptor to release arachidonic acid and downstream pro-inflammatory eicosanoids leading to hippocampal dysfunction, as we have observed in hippocampal dependent tasks in our model at chronic time points (Oestvang et al., 2011; Mouzon et al., 2014, 2018a,b). The differential effects in the cortex and hippocampus also seem to implicate possible changes in the net hydrolysis of PC and PE by phospholipase (PL) enzymes such as PLA_2_ and PLD following TBI in the hippocampus and cortex. The reason behind these localized regional brain changes observed at different time points in the hippocampus and cortex is currently unknown, but could indicate regional differences in lipid metabolism. It is also noteworthy in light of the diagnostic and therapeutic implications that we have focused our analyses on the whole cortex, and because our injury was performed on the mid-sagittal suture (localized to the parietal cortex), it is also possible that there might be an existence of a different lipid profile in different cortical regions, of which we are unable to determine in this current study.

Intriguingly, in a previous study examining wild-type mice exposed to the same experimental injury paradigm, a significant increase in these phospholipids (PC and PE) was also observed at acute time points post-injury in the cortex (Ojo et al., 2018). However, this was also accompanied by a significant increase at chronic time points (6–12 months) post-injury (Ojo et al., 2018). The reason behind this differential effect is unknown, but it is possible that these differences may be related to the underlying signaling processes activated by human tau in a murine background that normally expresses 3 repeat tau isoforms. Our previous behavioral and histopathological examination of animals appeared to indicate similar outcomes in spatial learning and memory, including glial and axonal pathology in both WT and hTau experimental models of repetitive mTBI (Mouzon et al., 2012, 2014, 2018a,b; Ojo et al., 2018). A closer assessment of other biochemical events will be required to address the differential effects exerted by the introduction of human vs. murine tau background and its impact on phospholipid metabolism, signaling and transport, and other lipid parameters such as small dense Low Density Lipoprotein, oxidized Low Density Lipoprotein, Apolipoproteins etc. Further studies will also be required to examine the consequences of early phospholipid changes on the integrity and behavior of neurons, glia and cerebrovascular cell function.

Our findings reported here are consistent with the human literature, whereby an immediate and significant increase in phospholipid levels in the CSF has been shown in patients exposed to severe TBI (Kay et al., 2003). In other mouse models of severe TBI, similar elevations in both phospholipids and their metabolites have likewise been demonstrated in the hippocampus and cortices of rodents following CCI at acute and sub-acute time points, respectively (Homayoun et al., 1997, 2000; Abdullah et al., 2014). Explanations for the immediate rise in phospholipid levels after TBI are unknown but could be attributed to repair of damaged phospholipid membranes and an early response to injury. Dysregulation in metabolic and catabolic processing of phospholipids and dysfunction in peroxisome mediated synthesis of phospholipids are possible mechanisms, but further biochemical investigations and a whole scale systemic approach are required to confirm this. It is known that blood brain barrier perturbations and reduction in cerebral perfusion is an early event following TBI (Amen et al., 2016) and this may contribute toward early and transient deleterious effects on brain lipid transport mechanism. It is also noteworthy that early increases in total PC and PE seem to resolve very early in our model, however, the consequences of the early events triggered by this rise is unknown. Phospholipids can be converted into downstream and bioactive lipids which can activate a complex downstream cascades of events that can be reparative or detrimental to cellular function. We tried to partially address this question herein, by examining the levels of PE and PC derivatives in our models, including the degree of saturation of different phospholipids.

We observed changes in etherPE (and etherPC) in the cortex and hippocampus at acute time points post-injury in our model. These lipids, particularly plasmalogens [etherPE (38:6); (40:4); (40:5); (40:6)] that contain O-alk-1-enyl at the sn1 position, generally contain more PUFA and play a role in cholesterol esterification and maintenance of myelin surrounding axonal nodes (Gorgas et al., 2006; Mankidy et al., 2010). Plasmalogens specifically play a vital role in vesicle formation and membrane fusion and therefore may also affect neurotransmitter release following TBI (Lohner, 1996). Over production of these lipid species could therefore have damaging consequences in neuronal signaling and may lead to dysfunction in neurotransmitter systems ubiquitous to the brain, such as glutamatergic systems. Glutamate excitotoxicity has been previously reported in TBI models *in vivo* (Yi and Hazell, 2006), and changes in etherPE, amongst other factors, may contribute to this biochemical event following injury.

In our TBI model we also observed a significant increase in sphingomyelin in the cortex and hippocampus at an early time point post-injury. We previously showed similar changes at acute (but also chronic) time points post-injury in wild type mice within the hippocampus of animals exposed to the same experimental r-mTBI paradigm (Ojo et al., 2018) and also in a CCI model (Abdullah et al., 2014). Sphingomyelin is a type of sphingolipid found in cell membranes and is associated with lipid microdomains or lipid rafts which influence cellular processes such as membrane sorting, trafficking and cell polarization (Wenk, 2010; Kosicek and Hecimovic, 2013). Sphingomyelin is also found in the membranous myelin sheath that surrounds some nerve cell axons. Axons become sheared following repeated mTBI, and this can be accompanied by disruptions and irregularities in myelin ensheathment of axons (Ojo et al., 2015). Increase in sphingomyelin may therefore indicate the activation of reparative processes involving membrane resorting, cellular trafficking and remyelination of damage axons. Additionally, sphingomyelin can also be metabolized to give rise to ceramides and other potentially toxic bioactive lipid species involved in apoptotic signaling. Whether this is a consequence of the increase in sphingomyelin in our model remains unknown, further measurement of downstream bioactive lipids will be need to elucidate this.

We examined the degree of saturation of fatty acids for each brain phospholipid species that we investigated in our mouse model and found an increase in SFA, MUFA, and PUFA levels in the cortex and hippocampus mainly at early time points (24 h and 3 months) post-injury for most of the phospholipid species. Degree of fatty acid saturation can influence the function of fatty acids in a variety of ways which can impact on neuronal and brain function. High intake of SFAs such as palmitic and stearic acid for example has been associated with increased risk for cardiovascular and neurodegenerative diseases, with evidence indicating increased tau hyperphosphorylation and increased expression of the amyloid beta generating enzyme, BACE1 (Patil et al., 2008). The source of this increase in SFA in our model is unknown (as all animals were on the same diet), but could be attributed to several reasons, such as: a lower capacity of neurons to metabolize SFAs, an increase in their synthesis in the brain or altered transport mechanisms into the brain that is TBI dependent (Spector, 1988a,b).

Monounsaturated fatty acids (MUFA) are a common part of Mediterranean diets and have long been associated with neuroprotective properties (Carrillo et al., 2012). In the brain the main MUFA is oleic acid, and it is found in neuronal membranes and in high levels in myelin (Bazinet and Laye, 2014). *In vivo* and *in vitro* studies have reported oleic acid’s ability to inhibit oxidative stress, promote anti-inflammatory/neurotropic support and mitigate AD pathology (Medina and Tabernero, 2002; Amtul et al., 2011; Carrillo et al., 2012). Human studies examining the CSF of patients exposed to TBI corroborate our findings, and confirm a significant increase in oleic acid 48 h after injury (Pilitsis et al., 2003). The increase in oleic acid observed at acute time points could signal an attempt by the brain to repair itself following TBI, and this is partly supported by the lack of overt histopathological changes in the cortex at sub-acute time points following injury in our experimental model (Mouzon et al., 2012, 2014). The source of increase in MUFA’s especially at an acute time point may be directly related to the desaturation of brain SFA’s which was also increased at this time point. Transit into the brain from the periphery as a result of transient disruptions to the BBB is also another plausible explanation.

The two most predominant PUFAs in the brain are omega-6 arachidonic acid (AA) and omega-3 docosahexaenoic acid (DHA). Arachidonic acid and docosahexaenoic acid each make up approximately 10% of the total fatty acids within brain phospholipids (Bazinet and Laye, 2014). In this study, within the cortex we reveal a significant increase in AA containing PI, PC and LPC at early time points post injury, and this was accompanied by a reduction in DHA levels for PI and LPC at 3 months post-injury. A significant increase in the AA to DHA ratio for PE, PC, and PI was also shown at the earlier time points post-injury in the cortex. We have previously reported a trend toward an increase in the AA to DHA ratio in the cortex of a similar TBI paradigm in wild-type mice at 3 and 6 months post-injury (Ojo et al., 2018) and also in the cortex following a single severe CCI TBI (Abdullah et al., 2014). In the hippocampus, we observed a significant increase in both AA and DHA levels for PC and LPC at the 24 h and 9 months post-injury time points; while the ratio of AA to DHA was increased for PC and PE species. These identified regional differences suggest that the molecular profile of TBI-induced events in the hippocampus and cortex may follow a different pathway, with a specific role played by certain phospholipid species in localized brain regions; but how this impacts on the pathogenesis of TBI is currently unknown. Human studies, examining CSF of patients exposed to TBI, also demonstrate an increase in PUFA and arachidonic acid 1 week after insult and this was associated with a worse outcome using the Glasgow Outcome Scale (Pilitsis et al., 2003). Arachidonic acid is a mediator lipid derived from long chain PUFAs; it is a precursor for downstream end mediators such as eicosanoids and leukotrienes that are produced in copious amounts by activated microglial cells in the brain, and are main drivers of pro-inflammatory responses and vascular permeability (Wenk, 2010). Decosahexaenoic acid which comprises 97% of the brain omega-3 long chain PUFAs is a precursor for end metabolites such as resolvins and protectins which have a potent anti-inflammatory role even at low nanomolar concentrations, including protection against oxidative stress, apoptosis and stimulation of neurite outgrowth and proliferation (Adibhatla and Hatcher, 2007; Ariel and Serhan, 2007; Bazinet and Laye, 2014). The secondary injury phase of TBI involves axonal injury mediated by shearing of axons, which causes damage to neuronal lipid rich membranes resulting in influx of calcium ions, depolarization of neurons and glutamate excitotoxicity. Such biochemical cascades can activate inflammatory processes leading to priming of glial cells and release of neurotoxic lipid metabolites, pro-inflammatory cytokines and free radical species (Bailes and Patel, 2014). This is partly supported by the evidence from our studies implicating an imbalance in AA to DHA ratio in our model. Increase in AA levels can result in conversion to eicosanoids by cyclooxygenase and lipoxygenase, which mediate pro-inflammatory processes, resulting in persistent activation of glial cells which express receptors that are sensitive to these bioactive lipids, thus resulting in a self-perpetuating cycle of tissue damage. Parallel reductions in DHA levels will also result in a decrease in their ability to yield bioactive lipid metabolites, such as protectin (neuroprotectin D1, resolvin), that can resolve inflammation and stimulate neurotrophic support (Bailes and Patel, 2014). Persistent glial activation and neuroinflammation in the white matter is a prominent feature of our model. We have yet to investigate the direct consequences of these changes in PUFAs in our model, and further work is planned to explore these possibilities, particularly focusing on downstream AA and DHA pathways. Thus from our findings it appears that dietary supplementation with DHA or mitigation of downstream AA eicosanoid pathways, especially at a therapeutic window within 3 months post injury where we observed most changes in our model, may offer a novel therapeutic approach to stimulate the brains DHA dependent neuroprotective mechanisms. Beneficial effects on neuroinflammation and behavioral outcomes have been previously shown in an animal model of concussion (acceleration and deceleration) with DHA supplementation (Bailes and Mills, 2010).

In this study we have conducted a comprehensive assessment of lipid profiles following TBI in the hTau mouse model over a longitudinal time scale. Our data indicate a significant increase at early time points post-injury in phospholipids, and elevations in the Omega-6 to Omega-3 fatty acid ratio. We propose that targeting these phospholipids and their bioactive metabolites offers a novel therapeutic approach in TBI, especially during the optimal therapeutic window for recovery of the normal lipid profile. Further studies using human samples (postmortem tissue, ISF/CSF, or plasma at different staging of pathogenesis) will be needed to translate our preclinical findings into the heterogeneous human TBI population. Moreover, additional investigations of how these brain specific changes may influence blood lipid profiles will be vital, as they may help to identify putative peripheral biomarkers of TBI, especially at early (acute to sub-acute) time points post-injury.

## Author Contributions

FC and MM conceived the project. FC, JO, MA, and JE directed the project. FC, JO, MA, and LA planned the experiments in the whole study. JO, FC, LA, MA, and JE were involved in the preparation of the manuscript. FC, JO, and BM participated in the establishment of the animal models. MA performed the majority of experiments, supported by JO, PL, and LA. MA, JO, and LA participated in the analysis of the experimental data. All authors contributed to the manuscript.

## Conflict of Interest Statement

The authors declare that the research was conducted in the absence of any commercial or financial relationships that could be construed as a potential conflict of interest.

## Supplementary Material

Table S1 Phosphatidylinositol lipid species in the cortex of r-mTBI mice. Significant changes in individual species containing phosphatidylinositol after repetitive mTBI in hTau mice. Sample size for all groups across all time points is *n* = 4. All data represent mean μM per (10 mg) wet weight ±SEM. Individual molecular lipid species were quantified by liquid chromatography/mass spectrometry. Highlighted boxes show significantly regulated levels (*P* < 0.01) between repetitive-mTBI/sham mice based on mixed linear modeling regression analysis.

Table S2 Phosphatidylethanolamine lipid species in the cortex of r-mTBI mice. Significant changes in individual species containing phosphatidylethanolamine after repetitive mTBI in hTau mice. Sample size for all groups across all time points is *n* = 4. All data represent mean μM per (10 mg) wet weight ±SEM. Individual molecular lipid species were quantified by liquid chromatography/mass spectrometry. Highlighted boxes show significantly regulated levels (*P* < 0.01) between repetitive-mTBI/sham mice based on mixed linear modeling regression analysis.

Table S3 Lysophosphatidylethanolamine lipid species in the cortex of r-mTBI mice. Significant changes in individual species containing lysophosphatidylethanolamine after repetitive mTBI in hTau mice. Sample size for all groups across all time points is *n* = 4. All data represent mean μM per (10 mg) wet weight ±SEM. Individual molecular lipid species were quantified by liquid chromatography/mass spectrometry. Highlighted boxes show significantly regulated levels (*P* < 0.01) between repetitive-mTBI/sham mice based on mixed linear modeling regression analysis.

Table S4 Phosphatidylcholine lipid species in the cortex of r-mTBI mice. Significant changes in individual species containing phosphatidylcholine after repetitive mTBI in hTau mice. Sample size for all groups across all time points is *n* = 4. All data represent mean μM per (10 mg) wet weight ±SEM. Individual molecular lipid species were quantified by liquid chromatography/mass spectrometry. Highlighted boxes show significantly regulated levels (*P* < 0.01) between repetitive-mTBI/sham mice based on mixed linear modeling regression analysis.

Table S5 Sphingomyelin lipid species in the cortex of r-mTBI mice. Significant changes in individual species containing sphingomyelin after repetitive mTBI in hTau mice. Sample size for all groups across all time points is *n* = 4. All data represent mean μM per (10 mg) wet weight ±SEM. Individual molecular lipid species were quantified by liquid chromatography/mass spectrometry. Highlighted boxes show significantly regulated levels (*P* < 0.01) between repetitive-mTBI/sham mice based on mixed linear modeling regression analysis.

Table S6 Phosphatidylethanolamine lipid species in the hippocampus of r-mTBI mice. Significant changes in individual species containing phosphatidylethanolamine after repetitive mTBI in hTau mice. Sample size for all groups across all time points is *n* = 4. All data represent mean μM per (5.5 mg) wet weight ±SEM. Individual molecular lipid species were quantified by liquid chromatography/mass spectrometry. Highlighted boxes show significantly regulated levels (*P* < 0.01) between repetitive-mTBI/sham mice based on mixed linear modeling regression analysis.

Table S7 Lysophosphatidylethanolamine lipid species in the hippocampus of r-mTBI mice. Significant changes in individual species containing lysophosphatidylethanolamine after repetitive mTBI in hTau mice. Sample size for all groups across all time points is *n* = 4. All data represent mean μM per (5.5 mg) wet weight ±SEM. Individual molecular lipid species were quantified by liquid chromatography/mass spectrometry. Highlighted boxes show significantly regulated levels (*P* < 0.01) between repetitive-mTBI/sham mice based on mixed linear modeling regression analysis.

Table S8 Phosphatidylcholine lipid species in the hippocampus of r-mTBI mice. Significant changes in individual species containing phosphatidylcholine after repetitive mTBI in hTau mice. Sample size for all groups across all time points is *n* = 4. All data represent mean μM per (5.5 mg) wet weight ±SEM. Individual molecular lipid species were quantified by liquid chromatography/mass spectrometry. Highlighted boxes show significantly regulated levels (*P* < 0.01) between repetitive-mTBI/sham mice based on mixed linear modeling regression analysis.

Table S9 Lysophosphatidylcholine lipid species in the hippocampus of r-mTBI mice. Significant changes in individual species containing lysophosphatidylcholine after repetitive mTBI in hTau mice. Sample size for all groups across all time points is *n* = 4. All data represent mean μM per (5.5 mg) wet weight ±SEM. Individual molecular lipid species were quantified by liquid chromatography/mass spectrometry. Highlighted boxes show significantly regulated levels (*P* < 0.01) between repetitive-mTBI/sham mice based on mixed linear modeling regression analysis.

Table S10 Sphingomyelin lipid species in the cortex of r-mTBI mice. Significant changes in individual species containing sphingomyelin after repetitive mTBI in hTau mice. Sample size for all groups across all time points is *n* = 4. All data represent mean μM per (5.5 mg) wet weight ±SEM. Individual molecular lipid species were quantified by liquid chromatography/mass spectrometry. Highlighted boxes show significantly regulated levels (*P* < 0.001) between repetitive-mTBI/sham mice based on mixed linear modeling regression analysis.
