# Supplementary figures and images for: Disruption in Brain Phospholipid Content in a Humanized Tau Transgenic Model Following Repetitive Mild Traumatic Brain Injury
Source: Front Neurosci. 2018 Dec 4;12:893. doi: 10.3389/fnins.2018.00893 (PMC6288299; doi:10.3389/fnins.2018.00893)

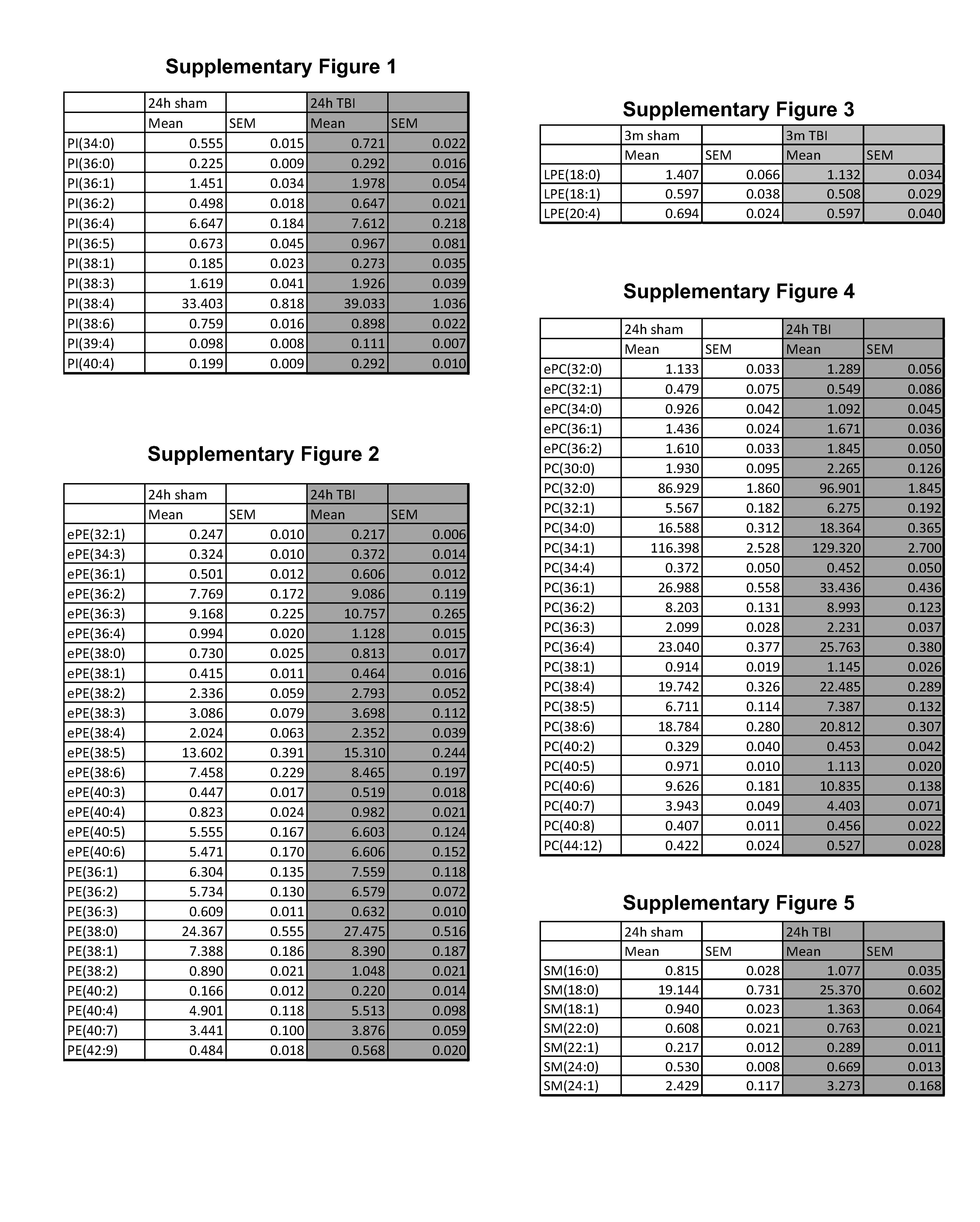

Supplement: Table S1 — Phosphatidylinositol lipid species in the cortex of r-mTBI mice. Significant changes in individual species containing phosphatidylinositol after repetitive mTBI in hTau mice. Sample size for all groups across all time points is n = 4. All data represent mean μM per (10 mg) wet weight ±SEM. Individual molecular lipid species were quantified by liquid chromatography/mass spectrometry. Highlighted boxes show significantly regulated levels (P < 0.01) between repetitive-mTBI/sham mice based on mixed linear modeling regression analysis. [file Image_1.JPEG]

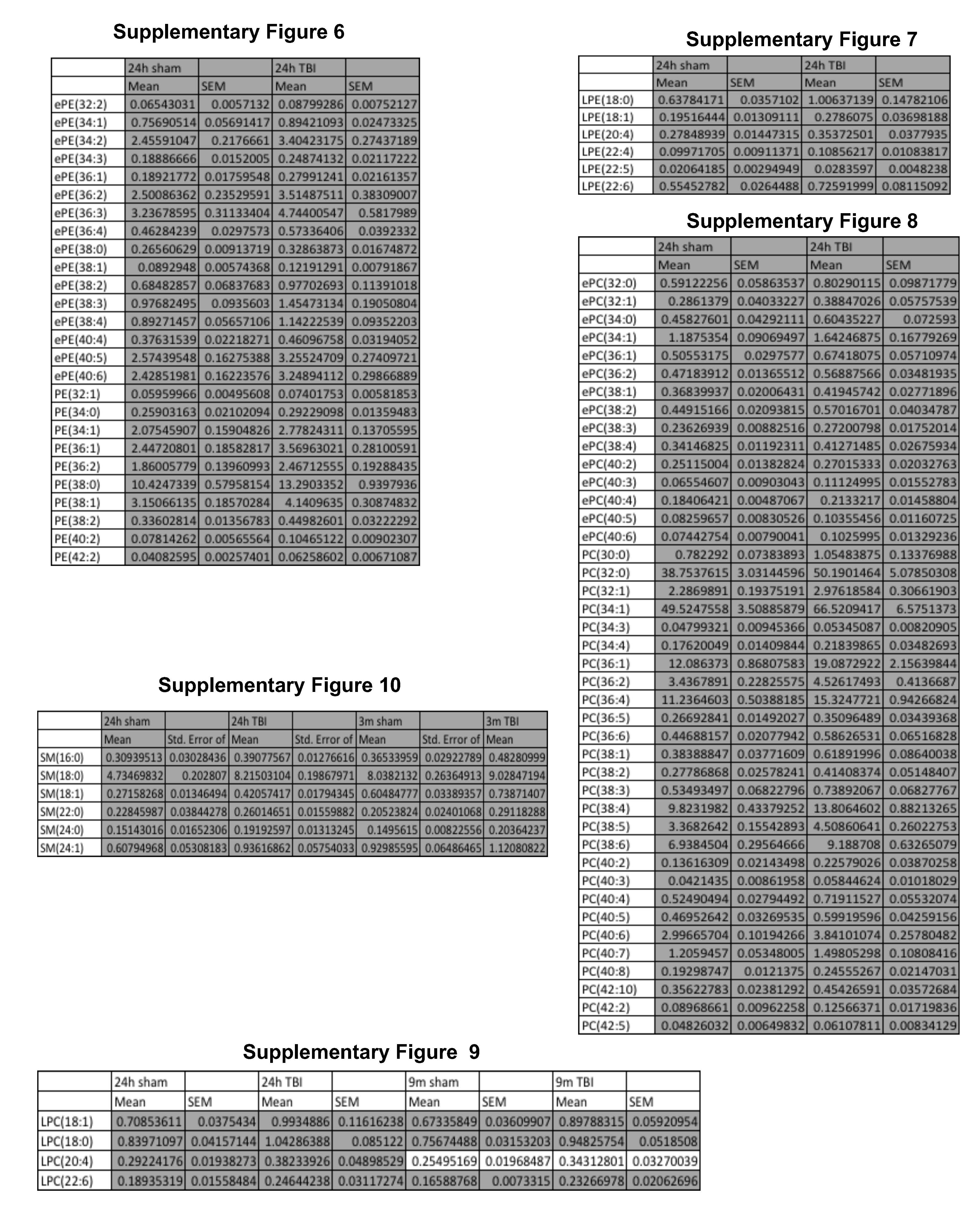

Supplement: Table S6 — Phosphatidylethanolamine lipid species in the hippocampus of r-mTBI mice. Significant changes in individual species containing phosphatidylethanolamine after repetitive mTBI in hTau mice. Sample size for all groups across all time points is n = 4. All data represent mean μM per (5.5 mg) wet weight ±SEM. Individual molecular lipid species were quantified by liquid chromatography/mass spectrometry. Highlighted boxes show significantly regulated levels (P < 0.01) between repetitive-mTBI/sham mice based on mixed linear modeling regression analysis. [file Image_2.JPEG]
